# Genome‐Wide Association Analysis of Hippocampal Neuroplasticity as an Indicator of Stress Responsiveness in Laying Hens (*Gallus gallus domesticus*)

**DOI:** 10.1002/age.70202

**Published:** 2026-09-10

**Authors:** Lindsay Neuert, Vern Osborne, Elena A. Armstrong, Fábio Pértille, Carlos Guerrero Bosagna, Timothy Boswell, Tom V. Smulders, Christine F. Baes, Bayode O. Makanjuola

**Affiliations:** ^1^ Department of Animal Biosciences, Centre for Genetic Improvement of Livestock University of Guelph Guelph Ontario Canada; ^2^ School of Psychology Newcastle University Newcastle upon Tyne UK; ^3^ Department of Organismal Biology, Physiology and Environmental Toxicology Uppsala University Uppsala Sweden; ^4^ School of Natural and Environmental Sciences Newcastle University Newcastle upon Tyne UK

**Keywords:** genome‐wide association study, hippocampus, laying hen, neuroplasticity, stress response

## Abstract

Environmental stressors in commercial poultry systems can negatively affect bird welfare, although individuals vary considerably in their responses. Neuroplasticity within the hippocampus, measured through the density of doublecortin‐positive (DCX^+^) neurons, provides a potential biomarker of stress experience in laying hens. However, the genetic basis underlying variation in this biomarker remains poorly understood. A total of 42 H&N and Hy‐Line Brown hens housed in a multitier free range and enriched cage system, respectively, were genotyped using Genotyping by Sequencing, yielding over 200 000 SNP markers after initial filtering. Hippocampal tissue sections were immunostained for DCX to quantify the density of highly plastic neurons. A genome‐wide association analysis identified 19 genomic regions across eight chromosomes within the top 1% of windows explaining the greatest proportion of genetic variance in the neuroplasticity phenotype. Within ±100 kb of these regions, 39 annotated genes were identified, several of which are involved in cellular regulation and genetic information processing pathways. Notably, *PIK3R6*, *VPS37D*, *STX1A*, *BAZ1B*, *HGH1*, *MAF1*, *MAPK15*, and *PIT54* emerged as positional candidate genes potentially contributing to variation in stress responsiveness. These findings provide preliminary insight into the genetic architecture of hippocampal neuroplasticity in laying hens and highlight candidate genes that may contribute to individual differences in stress response, with potential implications for breeding strategies aimed at improving poultry welfare.

## 1 Introduction

Exposure to stress could serve as a significant factor that contributes to the loss of function and reduced productivity of all organisms. Prolonged exposure to environmental stressors is associated with a high degree of stress response and this is an area of concern for the poultry industry due to the negative impact it has on body weight, egg production, and the overall welfare of the birds (McEwen and Seeman 1999; Shini et al. 2010). Therefore, efforts to mitigate the negative exposure of environmental stressors to enhance the overall welfare of poultry are one of the goals of producers. To boost positive welfare in poultry, genetic selection for a healthy affective response and improving the production environment are two possible approaches to be considered (Shini et al. 2010). Recent research has shown that housing systems could be responsible for differences in physiological and behavioral parameters such as secretory and plasma immunoglobulin A, feather corticosterone and tonic immobility between birds due to increased fear, chronic fear responses, and increased anxiety (Campbell et al. 2022). The responses of birds in these systems could elicit negative affective experiences. However, there are still individual differences that exist in these housing groups. Additionally, genetic marker differences between high‐stress and low‐stress Japanese quail have been reported to cause observed phenotypic differences (Shumaker et al. 2021). Therefore, acquiring information on genetic markers can give insights into heritable traits and provides implications for sustainable and efficient farming. Genetic variations within and between populations contribute to the observed phenotypic diversities. Hence, identifying single nucleotide polymorphisms (SNP) associated with lower stress responsivity and their biological function may allow a greater understanding of their impact on gene function and phenotype. SNP are often used as genetic markers in population genetic studies (Syvänen 2001).

The hippocampus has been implicated in the affective experience through its inhibitory feedback on the hypothalamic–pituitary–adrenal (HPA) axis, which is a central component of the stress response system (Larosa and Wong 2022). The rodent hippocampus can be spatially segregated into dorsal and ventral regions, with the dorsal region playing a vital role in cognitive functions, while the ventral area is important in affective processes (Fanselow and Dong 2010). In birds as well, this functional subdivision of the hippocampus has been proposed, with the rostral hippocampal formation more involved in cognitive processes and the caudal subdivision more involved in affective processes (Smulders 2017; Madison et al. 2024).

In rats and mice, studies have suggested that positive and negative cumulative affective experiences can be quantified by adult hippocampal neurogenesis. The highest levels of neurogenesis are shown from multiple positive experiences (Fabel et al. 2009), intermediate levels for positive and negative experiences (Veena et al. 2009; Zheng et al. 2017), and low levels for more negative experiences (Romero‐Grimaldi et al. 2015). It is important to note that affective experiences are a combination of external environmental factors and the animal's response to those factors. Some of the individual differences in responses of the animals to the external factors could be attributed to genetic influence. However, there is a lack of knowledge on specific individual genetic differences that lead to the manifestation of these cumulative affective experiences.

Studies have used the plasticity marker doublecortin (DCX) to identify and quantify new hippocampal neurons in a mixed population of neurons (Rao and Shetty 2004). However, there is still inconclusive evidence whether DCX exclusively labels new neurons or that it also includes neurons with high plasticity, especially in birds (Balthazart and Ball 2014a, 2014b; Vellema et al. 2014). In poultry, DCX‐expressing neurons in the hippocampus have been used as a biomarker of chronic stressful experience. The density of DCX^+^ neurons has been observed to decrease in response to unpredictable stress (Gualtieri et al. 2019), severe keel bone fractures (Armstrong et al. 2020), and poor body condition (Armstrong et al. 2022). DCX^+^ neurons were classified by their morphology as bipolar/fusiform (spindle‐shaped, with two primary processes, typically representing an earlier stage of neuronal maturation) or multipolar (possessing three or more processes, typically representing a later maturation stage), and were quantified separately within the rostral and caudal hippocampal subregions. This yielded four phenotypes referred to throughout this article by their abbreviations: caudal bipolar (CB), caudal multipolar (CM), rostral bipolar (RB), and rostral multipolar (RM). We hypothesized that individual variation in hippocampal DCX^+^ neuron density in laying hens may have a partial genetic basis, and that a genome‐wide association approach would identify candidate genomic regions and genes associated with this stress‐related trait. Therefore, the objectives of this study were to (1) use a genome‐wide association study to identify genomic regions that are associated with densities of DCX^+^ bipolar and multipolar neurons in the rostral and caudal region of the laying hen hippocampus, and (2) annotate the identified regions to determine their biological function.

## 2 Materials and Methods

### 2.1 Ethical Statement

Animal samples used in this study were provided by Newcastle University and ethical approval was given by the Animal Welfare and Ethical Review Body at Newcastle University (Project ID #702). All methods were performed in accordance with the relevant guidelines and regulations. RSPCA‐Assured standards were followed during the housing and managing of the animals, while farming, DEFRA guidelines were adhered to, and while at the university, the Home Office Code of Practice was followed (Armstrong et al. 2022). A Home Office Schedule 1 was used for the method of euthanasia. The study reported is in accordance with ARRIVE guidelines.

### 2.2 Study Population

The study used H&N and Hy‐Line brown hens ( *Gallus gallus* *domesticus*) that were housed respectively in a multitier housing system with access to an outdoor range or in an enriched cage system. More details on their housing can be found in Armstrong et al. (2022). Briefly, the study collected hens with external indicators of physical body conditions (good or poor) from multitier and enriched cage housing systems. Criteria included body size, feather coverage, and redness of the comb, with high criteria levels indicating good condition. At the time of sampling, H&N hens were 65 weeks of age and Hy‐Line hens were 66 weeks of age. Over 4 days, 12 birds were selected each day (total *n* = 48) (3 from the H&N/multitier and good condition; 3 from the H&N/multitier and poor condition; 3 from the Hy‐Line/enriched cage and good condition; 3 from the HyLine/enriched cage and poor condition). More details on the study population are presented in Armstrong et al. (2022). Of these hens, the phenotypic records available for the purpose of this study consisted of 11 from the H&N/multitier and good condition, 10 from the H&N/multitier and poor condition, 10 from the Hy‐Line/enriched cage and good condition, 11 from the Hy‐Line/enriched cage and poor condition (*n* = 42).

### 2.3 Tissue Collection and Processing

The birds were weighed and euthanized according to a schedule that alternated between the housing type/strain of the hens and body condition for the hens. Blood samples were collected for DNA analysis. The brain was removed and prepared for immunohistochemical analysis in a way that was balanced within groups of body condition and from each of the different housing systems (Armstrong et al. 2022).

An antibody to doublecortin (DCX) was used to stain the hippocampal formation to quantify immature neurons. Free‐floating sections from hens underwent staining in six batches, each comprising tissue from eight birds (six batches × eight birds to yield samples from 48 birds, in which 42 had viable phenotypic information). Per batch, these eight birds consisted of two H&N/multitier and good condition, two from the H&N/multitier and poor condition, two from the Hy‐Line/enriched cage and good condition, and two from the Hy‐Line/enriched cage and poor condition. Staining followed the protocol detailed in Armstrong et al. (2020). The DCX immunostaining protocol has been previously validated. Inter‐assay technical variability, quantified as the coefficient of variation (CV) in DCX^+^ cell counts across repeated staining batches, has been previously described and validated in Armstrong et al. (2020, 2022); as the phenotypic data used here were sourced directly from those studies, CV values are not recalculated or re‐reported in this article. The primary antibody was rabbit polyclonal to doublecortin (Cat# ab18723; Abcam, Cambridge, UK), incubated at a concentration of 1:1000 for 18 h at 4°C, and the secondary antibody incubation utilized biotinylated anti‐rabbit immunoglobulin G secondary antibody produced in goats (BA‐1000; Vector Laboratories, Newark, CA, USA) at concentration 1:500 for 120 min at room temperature (Armstrong et al. 2022). Cells were visualized using 3,3′‐diaminobenzidine (DAB) as the chromogen. Quantification was carried out without knowledge of the housing and body condition groups (Armstrong et al. 2022). DCX^+^ cells with multipolar and bipolar/fusiform morphologies were tallied independently, as detailed in Armstrong et al. (2020). The density of both cell morphologies was calculated separately for the rostral and the caudal hippocampus by dividing the number of cells counted of each morphology by the volume of tissue sampled. More details on the tissue processing, quantification of adult hippocampal plasticity, and data analysis are presented in Armstrong et al. (2022).

### 2.4 Genotype Data

Genomic DNA from the blood samples was extracted, digested with the PstI enzyme, and ligated to adapters containing barcodes to identify individual animals, using the Genotyping by Sequencing method as previously described (Pértille et al. 2016). Sequencing libraries were sent for quantification, clustering, and paired‐end sequencing on the Illumina NovaSeq 6000 platform, with a read length of 150 bp at the SNP&SEQ Technology Platform of SciLifeLab (Uppsala, Sweden).

Raw data analysis, genotyping, and SNP identification were conducted using the TASSEL 5.0 software (Bradbury et al. 2007) following its pipeline's default parameters with the chicken genome bGalGal1.mat.broiler.GRCg7b (GCA_016699485.1, NCBI) as the reference. Initial filtering criteria included retaining loci with a minimum coverage of 10% across taxa and a minor allele frequency > 0.01. Initially, a total of 211 075 SNP markers were identified from the raw data.

To improve genotype call rates and handle missing data, we applied a *k*‐nearest neighbors imputation (kNNi). A total of 10 number of neighbors (*k* = 10) was used for imputation. This method imputes missing values by finding the *k* observations from the neighboring non‐missing features and averages the values for the missing value (Troyanskaya et al. 2001). This imputation approach was implemented within TASSEL, using the LD‐kNNi algorithm, which leverages linkage disequilibrium (LD) structure to improve the accuracy of imputation. For LD, a total of 30 high LD sites were used to identify good predictors for each SNP. Subsequently, data preparation for downstream analysis and quality control measures were performed on the genotype data using PLINK v1.9 software (Chang et al. 2015). The quality control measure used includes the removal of non‐autosomal markers, SNPs with a minor allele frequency < 0.05 and SNP markers with a missing call rate > 0.10. After quality control measures, a total of 121 346 SNP genotype markers with an approximate average of 0.98 genotype call rate were retained for statistical analysis.

### 2.5 Statistical Analyses

The experimental unit was the individual hen (*n* = 42). To evaluate the association between regions of the genome and highly plastic neurons (bipolar and multipolar) in the hippocampus, the proportion of genetic variance explained by each SNP marker was calculated. Genome‐wide Complex Trait Analysis (GCTA) v1.94.1 software was used to estimate the genetic variance explained by all the SNP markers available. Specifically, in GCTA the MLMA method was employed (Yang et al. 2011). To estimate the proportion of genetic variance explained, the following linear model used was:

𝑦=Wα+𝑥𝛽+u+e

where ***y*** is a *n* × 1 vector of phenotypes from *n* individuals, **W** is a *n* × *c* matrix of fixed effects of body conditions (two levels; Good and Poor) and staining batches (six levels; 1–6), **α** is a *c* × 1 vector of fixed effect coefficients, ***x*** is a *q* × 1 vector of marker genotypes at the *q*th tested locus, 𝛽 is the effect size of the marker, ***u*** is a *n* × 1 vector of random polygenic effects, and ***e*** is a *n* × 1 vector of the error term. The assumption for the random effects are:

$$u\sim N\left(0,G\sigma_{g}^{2}\right)\;\text{and}\;e\sim N\left(0,I\sigma_{e}^{2}\right)$$

where **G** is the *n* × *n* genomic relationship matrix derived from the SNP markers, $\sigma_{g}^{2}$ is the polygenic additive variance, **I** is a *n* × *n* identity matrix and $\sigma_{e}^{2}$ is the residual variance.

To identify regions with the highest genetic variance explained, variances were calculated from overlapping sliding windows of 20 SNP. The top 1% of regions with the highest proportion of genetic variance explained were selected. The threshold for statistical significance was set at *p* < 0.05. Bonferroni correction was initially applied to assess genome‐wide significance; however, as no SNP effects reached the corrected threshold (Duggal et al. 2008), the proportion of genetic variance explained was used as the primary criterion for identifying candidate regions. Tendency was not defined or applied in these analyses.

### 2.6 Assignment of Identified Genomic Regions

Genomic regions were defined based on literature reports that strong linkage disequilibrium in chickens ranges from 10 to 150 kb (Rao et al. 2008; Qanbari et al. 2010). Ensembl was used to retrieve genes located within 100 kb upstream and downstream of the identified genomic regions explaining high proportion of genetic variance (Martin et al. 2023). The reference chicken genome assembly bGalGal.matbroiler.GRCg7b with the reference sequence accession GCA_016699485.1 was used. Kyoto Encyclopedia of Genes and Genomes (KEGG) was used to assign identified genes to metabolic and signaling pathways and to associated diseases (Kanehisa and Goto 2000); identified genes found in the KEGG database were referenced to the human genome and further investigated using the NCBI gene database. Gene Ontology (GO) term enrichment, comprising the biological process, cellular component, and molecular function branches, was conducted separately using the gprofiler‐official Python package, specifying *Homo sapiens* as the target species (Raudvere et al. 2019). Given that the gene list was selected based on variance explained and sample size constrains (*n* = 42), we used a less stringent filtering threshold (FDR < 0.1) to identify enriched biological processes. Both KEGG and GO enrichment analyses were referenced to the human genome due to a larger depth of information on *Homo sapiens* compared to *Gallus gallus* , further emphasizing the novelty of research on poultry stress response.

## 3 Results

The estimated SNP effects were not statistically significant after correcting for multiple testing, as detailed in Section 2.5 (Figure S1). Therefore, the proportion of genetic variance explained by SNPs was used to identify potential candidate SNPs associated with hippocampal DCX^+^ neural density. There were 19 identified regions spanning eight chromosomes with a high proportion of genetic variance explained relating to the density of highly plastic neurons in the hippocampus as presented in Table 1. A total of 39 different genes with known functions were found in or proximal to these regions, which spanned across Chromosomes 2, 9, 12, 18, 19, and 33. The genes and their locations that were identified with association to CB, CM, RB, or RM density are listed in Table 2.

**TABLE 1:** Identified regions on the genome with top 1% proportion of genetic variance explained regarding the density of the neuron type in the subregion of the hippocampus in Hy‐Line and H&N chickens.

Trait	Chromosome	Start position (bp)	End position (bp)	Effect size	Variance explained by a window of 20 SNP markers
CB	18	2464841	2473064	−27.59	0.18
	18	2484377	2489918	−206.78	0.22
	18	2506719	2506719	720.77	0.16
	19	704467	727567	−305.74	0.16
CM	18	2484377	2506723	−75.76	0.28
	19	704467	727555	−62.35	0.22
RB	2	148610822	148798234	−12.78	0.13
	9	4030271	4047242	−24.53	0.11
	18	2455441	2457190	−52.57	0.12
	18	2463597	2468974	−19.42	0.11
	18	2519401	2524267	69.81	0.11
	18	2524278	2529803	−16.58	0.1
	19	727555	727555	−173.46	0.1
	33	1089564	1118155	−3.84	0.11
RM	12	6652437	6794581	−26.44	0.17
	16	1143788	1147910	−20.49	0.18
	18	2457190	2457203	−7.87	0.15
	18	2463597	2468974	−16.42	0.16
	31	239012	563856	11.19	0.2

Abbreviations: CB, caudal bipolar; Chr, chromosome; CM, caudal multipolar; RB, rostral bipolar; RM, rostral multipolar.

**TABLE 2:** Location and associated traits of genes found inside, 100 kb upstream, or 100 kb downstream regions with a high proportion of genetic variance explained in Hy‐Line and H&N chickens.

Trait	Start position (bp)	End position (bp)	Chromosome	Gene
CB, CM, RB	2582753	2605437	18	*PIK3R6*
CB, CM	611675	613002	19	*VPS37D*
CB, CM	618008	619285	19	*DNAJC30*
CB, CM	618754	624163	19	*WBSCR22*
CB, CM	623897	671557	19	*STX1A*
CB, CM, RB	677984	689028	19	*MKS1*
CB, CM, RB	692807	697245	19	*BCL7B*
CB, CM, RB	697636	701987	19	*TBL2*
CB, CM, RB	702127	717312	19	*MLXIPL*
CB, CM, RB	726965	728781	19	*ANGPT1L*
CB, CM, RB	745493	794545	19	*BAZ1B*
CB, CM, RB	797536	799813	19	*FZD9*
RB	148537963	148566708	2	*HGH1*
RB	148570511	148590058	2	*MAF1*
RB	148590419	148636483	2	*SHARPIN*
RB	148647472	148679868	2	*RECQL4*
RB	148679892	148687605	2	*LRRC14*
RB	148689718	148695856	2	*C8orf82*
RB	148700991	148787636	2	*ARHGAP39*
RB	148839874	148857045	2	*CCDC166*
RB	148856791	148876628	2	*MAPK15*
RB	4004998	4008805	9	*CRYGS*
RB	4010829	4019192	9	*TBCCD1*
RB	4018632	4033775	9	*DNAJB11*
RB	4037842	4039914	9	*PROCR*
RB	4038030	4045514	9	*PFKL*
RB	4047531	4054037	9	*CFAP410*
RB	4054357	4072805	9	*TRA2B*
RB	4079191	4096152	9	*IGF2BP2*
RB	4098182	4112345	9	*SENP2*
RB	4123696	4130671	9	*AMOTL2*
RM	6569002	6620225	12	*FAM120A*
RM	6650796	6757212	12	*WNK2*
RM	6793715	6811552	12	*NINJ1*
RM	6838157	6867611	12	*SUSD3*
RM	1069617	1069769	16	*5_8S_rRNA*
RM	1129255	1129407	16	*5_8S_rRNA*
RM	1157750	1157902	16	*5_8S_rRNA*
RM	1185692	1185844	16	*5_8S_rRNA*
RM	241488	248814	31	*PIT54*
RM	263621	280351	31	*CD163L*

*Note:* Gene name is written in italic.

Abbreviations: CB, caudal bipolar; CM, caudal multipolar; RB, rostral bipolar; RM, rostral multipolar.

For the biological pathway analysis, KEGG Brite classification of the genes within or near the identified regions is summarized in Table 3. There were 13 identified genes that were not annotated in the KEGG Brite database and subsequently were omitted from this table. GO enrichment analysis identified one significant biological process enriched in the genes explaining the top 1% of genetic variance: the Wnt signaling pathway (*GO:0016055*, *p* = 0.0386). Six genes (*MKS1*, *BCL7B*, *FZD9*, *SENP2*, *AMOTL2*, and *WNK2*) were enriched in this pathway, defined as “the series of molecular signals initiated by binding of a Wnt protein to a frizzled family receptor on the surface of a target cell and ending with a change in cell state.”

**TABLE 3:** KEGG Brite classification system of known genes found inside, 100 kb upstream, or 100 kb downstream regions with a high proportion of genetic variance explained in Hy‐Line and H&N chickens. Genes classified based on the human organism.

KEGG Brite	Genes
Enzymes	*WBSCR22*, *PFKL*, *WNK2*, *MAPK15*, *SENP2*, *RECQL4*
Membrane trafficking	*STX1A*, *PIK3R6*, *VPS37D*, *ARHGAP39*
Protein phosphatases and associated proteins	*PFKL*, *TRA2B*, *WNK2*
CD molecules	*CD163L*, *PROCR*, *FZD9*
Chromosome and associated proteins	*BCL7B*, *BAZ1B*
DNA repair and recombination proteins	*RECQL4*, *CFAP410*
Messenger RNA biogenesis	*SENP2*, *PFKL*
Chaperones and folding catalysts	*DNAJB11*, *DNAJC30*
Ubiquitin system	*SHARPIN*, *SENP2*
Protein kinases	*MAPK15*, *WNK2*
Domain‐containing proteins not elsewhere classified	*SUSD3*, *IGF2BP2*
Cilium and associated proteins	*TXNDC15*, *CFAP410*
Translation factors	*TBL2*
G protein‐coupled receptors	*FZD9*
Ribosome biogenesis	*WBSCR22*
DNA replication proteins	*RECQL4*
Spliceosome	*TRA2B*
Peptidases and inhibitors	*SENP2*
Exosome	*VPS37D*
Transcription factors	*MLXIPL*

*Note:* Gene name is written in italic.

**FIGURE 1:**
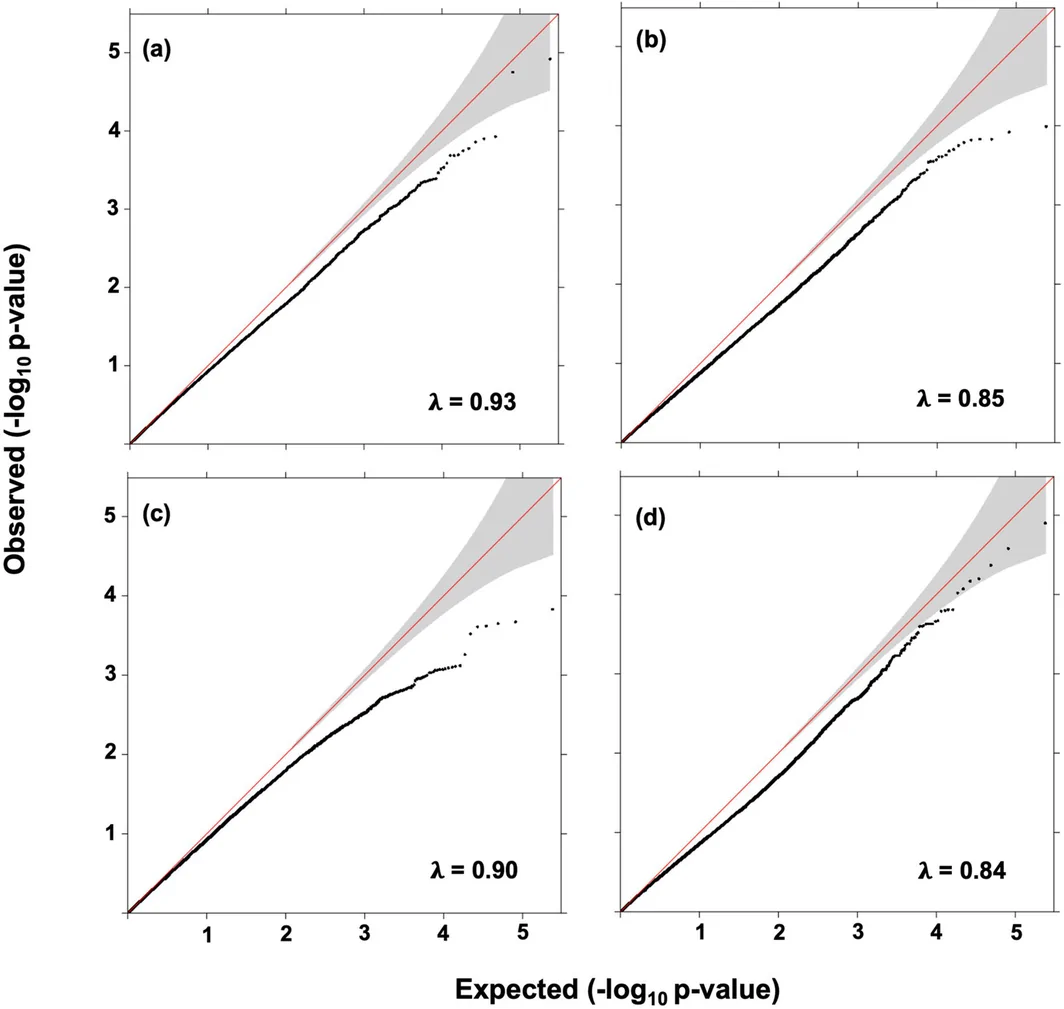
Four quantile‐quantile plots (a–d) displaying observed negative log_10_ *p* values on the *y*‐axis versus expected negative log_10_ *p* values on the *x*‐axis, from a genome‐wide association study for hippocampal neural density in laying hens. Panels a–d represent: (a) caudal bipolar, (b) caudal multipolar, (c) rostral bipolar, and (d) rostral multipolar.

**FIGURE 2:**
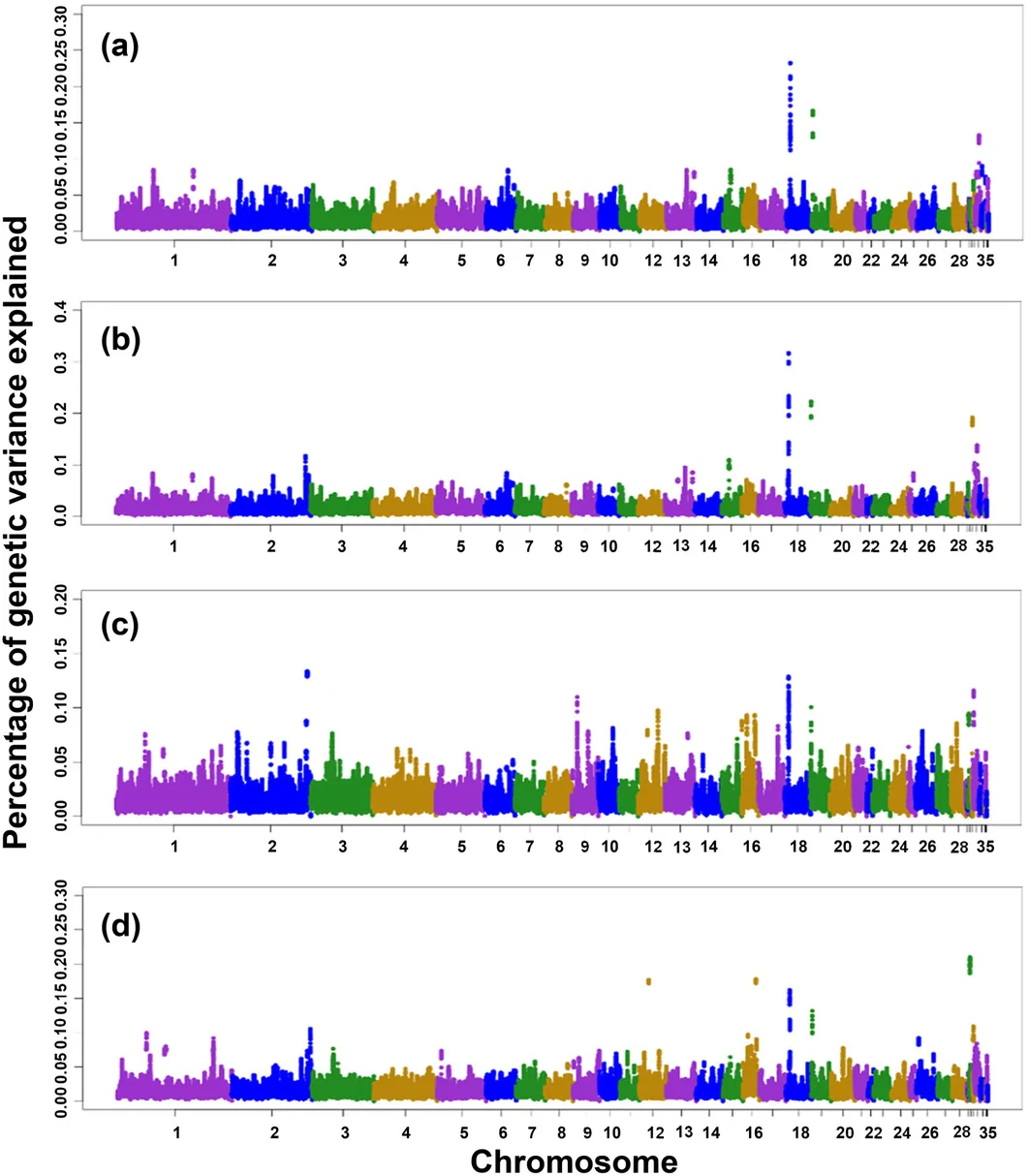
Manhattan plot of proportion of genetic variance explained by 20‐SNP windows across chromosomal positions for hippocampal neuron density in laying hens. Panels a–d represent: (a) caudal bipolar, (b) caudal multipolar, (c) rostral bipolar, and (d) rostral multipolar.

In total, four regions with a high proportion of genetic variance explained were identified through the GWAS of CB neurons. The quantile‐quantile plot and Manhattan plot for CB are presented in Figures 1a and 2a. Three regions were identified on Chromosome 18 and one on Chromosome 19. For CM, a total of two regions with a high proportion of genetic variance explained were identified. The corresponding quantile‐quantile plot and Manhattan plot for CM are presented in Figures 1b and 2b. One region was identified on Chromosome 18 and another on Chromosome 19.

A total of eight regions with a high proportion of genetic variance explained were identified through the GWAS of RB. The Manhattan plot depicting the proportion of genetic variance explained for RB is presented in Figures 1c and 2c. For RM, five regions with a high proportion of genetic variance explained were identified. The corresponding Manhattan plot for RM is presented in Figures 1d and 2d. These regions were located on Chromosomes 12, 16, 18, and 31.

The identified genes in this study were implicated in 61 KEGG pathways. Of these genes, three of the pathways were involved with two genes while the others corresponded to one gene. The ATP‐dependent chromatin remodeling pathway related to *BAZ1B* and *BCL7B*. *FZD9* and *STX1A* relate to the KEGG pathway of neurodegeneration relating to multiple diseases. The Wnt signaling pathway is related to *FZD9* and *SENP2*. Based on the KEGG database, eight (*VPS37D*, *DNAJC30*, *WBSCR22*, *BCL7B*, *TBL2*, *MLXIPL*, *BAZ1B*, *FZD9*) of the identified genes may be related to the plasticity of the caudal hippocampus (bipolar and multipolar neurons) and may play a role in Williams‐Beuren Syndrome.

## 4 Discussion

The densities of CB, CM, RB, and RM, as a biomarker of stress, may be associated with specific genes in laying hens. Across these four phenotypes, there were a total of 39 different genes found in the regions with a high proportion of genetic variance explained.

*PIK3R6* (phosphoinositide 3‐kinase gamma regulatory subunit 6) was associated with CB, CM, and RB density. *PIK3R6* encodes a subunit of the phosphoinositide‐3‐kinase (PIK3) complex, which is involved in leukocyte motility and immune signaling. PIK3R6 was also identified in a GWAS of high‐ and low‐stress lines of Japanese quail (Shumaker et al. 2021), which is consistent with a possible role in stress response, though this parallel should be interpreted cautiously given the modest sample sizes in both studies.

*VPS37D* (vacuolar protein sorting 37 homolog D) was associated with CB and CM density. The encoded protein is involved in vacuolar protein sorting and ubiquitin‐dependent membrane trafficking. Depletion of VPS37 has been shown to evoke cellular stress responses (Kolmus et al. 2021) and inflammatory responses and immunogenic cell death (Szymańska et al. 2020), raising the possibility that *VPS37D* contributes to a biological mechanism for coping with stress.

*STX1A* (syntaxin 1A) was associated with CB and CM density. *STX1A* encodes a nervous‐system‐specific protein that facilitates synaptic vesicle docking at the presynaptic membrane. *STX1A* knockout mice show blunted hypothalamic–pituitary–adrenal (HPA) axis activity, with reduced corticosterone and adrenocorticotropic hormone release at rest and after acute restraint stress (Fujiwara et al. 2011), suggesting a plausible, though indirect, mechanistic link to stress reactivity.

*BAZ1B* (bromodomain adjacent to zinc finger domain protein 1B) was associated with CB, CM, and RB density. *BAZ1B* encodes a bromodomain protein involved in chromatin‐dependent transcriptional regulation. *BAZ1B‐*knockout zebrafish display reduced stress response (Torres‐Perez et al. 2022), and upregulation of *BAZ1B* in the mouse nucleus accumbens has been associated with resilience to chronic social defeat stress (Sun et al. 2016), together pointing to a plausible role in stress regulation.

*HGH1* (HGH1 homolog) was associated with RB density. Knockout studies link *HGH1* to Hsp90 availability and heat‐shock signaling (Alford and Brandman 2018), and to stress‐granule formation under nutrient and heat stress in *S. cerevisiae* (Yang et al. 2011), with distinct roles reported under different stress conditions. These findings are broadly consistent with a possible role for *HGH1* in stress response, though this remains to be tested directly in this system.

*MAF1* (MAF1 homolog) was associated with RB density. *MAF1* encodes a leucine‐zipper transcription factor that can act as either a repressor or activator. Genes in the MAF family have been implicated in the human stress response, including modest induction by heat shock and heavy‐metal exposure (Suzuki et al. 2001), offering an indirect line of biological plausibility.

*MAPK15* (mitogen‐activated protein kinase 15) was associated with RB density. *MAPK15* promotes chromatin binding activity and, in a kinase‐dependent manner, is involved in ciliogenesis, protein trafficking, and autophagy. It has been reported to be upregulated under multiple stress conditions, including heat and oxidative stress (Singh et al. 2018).

*PIT54*, associated with RM density, encodes an antioxidant hemoglobin‐binding protein. Although *PIT54* expression was not altered by transportation stress in turkeys, it was proposed that a more intense stressor may be required to detect changes in its expression (Marques et al. 2016), leaving its relevance to stress response in this study inconclusive.

In this study, *PIK3R6*, *VPS37D*, *STX1A*, *BAZ1B*, *HGH1*, *MAF1*, *MAPK15*, and *PIT54* were identified as potential candidate genes associated with hippocampal DCX^+^ neural density, a biomarker for cumulative affective experience. These genes have been consistently implicated in stress responses across various studies, which adds weight to their potential significance in understanding how animals cope with and react to stressors. This suggests that these genes may play a role in how animals respond to stressors and how their affective experiences accumulate over time. Further research and validation will be necessary to fully understand the extent of their involvement in these processes, for example, through independent genotyping in larger cohorts, or targeted expression and functional studies for genes with existing functional data in other species (e.g., *BAZ1B*, *STX1A*).

Eight of the identified genes (*VPS37D*, *DNAJC30*, *WBSCR22*, *BCL7B*, *TBL2*, *MLXIPL*, *BAZ1B*, and *FZD9*), all associated with caudal rather than rostral hippocampal neuron density, fall within the region deleted in Williams‐Beuren Syndrome (WBS), a human microdeletion disorder on Chromosome 7 (Mariasina et al. 2022). Of particular relevance to this study, WBS is associated with a distinctive behavioral phenotype that includes heightened anxiety and atypical social affect (Osborne 2010), and comparable phenotypic features have been described in animal models carrying the corresponding deletion (VonHoldt et al. 2017). The consistent co‐occurrence of these genes among the caudal hippocampal candidates is therefore worth further investigation as a potential link between this gene cluster and affective/stress‐related processing in chickens, independent of the syndrome's other clinical features.

Genomic regions with high genetic variance explained in both CB and CM were identified on Chromosomes 18 and 19. The two regions in the CM group were within 100 kb of the regions found in the CB. The same genes associated with CM were the same as the ones identified regarding CB. This indicates that similar regions and therefore genes on Chromosomes 18 and 19 play a role in neuroplasticity in the caudal hippocampus, a biomarker for stress. In addition, it indicates that the two morphologies are most likely to be different stages in the differentiation of the same cell type (Boseret et al. 2007), and that stress affects this process early on, so both early (bipolar) and late (multipolar) stages are affected. Both RB and RM were associated with more widespread regions on the genome. This may indicate that these cells have different processes regulating their differentiation at different stages. Despite the diversity, the regions on Chromosome 18 were within 100 kb of each other across RB and RM. FDR was used to maximize the power to detect true positive associations while still controlling the proportion of false positives. To allow for the exploratory nature of this analysis with the smaller sample size, a less stringent threshold (FDR < 0.1) was adopted to allow the detection of biologically meaningful signals that might be missed under more conservative cutoffs (e.g., FDR < 0.05). This approach is commonly employed in studies with limited statistical power, where overly strict multiple‐testing corrections may obscure true associations of modest effect size (Hu et al. 2005). Importantly, the identified candidates were interpreted with caution and further evaluated in the context of prior biological knowledge and pathway enrichment to mitigate the risk of spurious findings. Due to the small sample size, considerations and caution should be taken when interpreting the results from this study regarding the novelty and sample size. There is a lack of research on identifying specific genes related to stress reactivity in poultry using the methodology followed in this study. To validate the association of the identified genes with stress, the same methodology should be employed in other independent studies investigating stress in chickens, as well as different species. Furthermore, future studies should employ a larger sample size to validate the results.

Overall, this study identified SNP markers with high genetic variance that are associated with DCX^+^ densities, which are potentially due to individual differences in responses to stress.

There were a number of genes known to be involved in stress responses, indicating that densities of DCX^+^ cells may be good indicators of individual experiences of stress. This supports the use of DCX^+^ neurons as biomarkers of welfare in laying hens and probably other animals. The findings provide a starting template for the basic understanding of the genetic architecture that underpins stress reactivity in laying hens, as measured by known biomarkers of hippocampal plasticity. Future independent research is warranted to validate the identified genes.

### 4.1 Limitations

This study provides an insight into the use of DCX^+^ neuron as a biomarker to assess the welfare of laying hens housed in multitier aviaries or enriched cages. However, certain limitations in this study may affect the interpretation of the findings. One primary limitation is the sample size used in this study, which reduced the statistical power to detect significant markers when correcting for multiple testing using Bonferroni threshold. Direct evaluation of candidate gene expression in hippocampal tissue was beyond the scope of this study and represents an important direction for future work.

## Author Contributions

**Lindsay Neuert:** formal analysis (lead), writing – original draft preparation (lead). **Vern Osborne:** writing – review and editing (equal). **Elena A. Armstrong:** conceptualization (equal), data curation (equal). **Fábio Pértille:** data curation (equal), resources (equal), writing – review and editing. **Carlos Guerrero Bosagna:** data curation (equal), resources (equal), writing – review and editing (equal). **Timothy Boswell:** conceptualization (equal). **Tom V. Smulders:** conceptualization (equal), writing – review and editing (equal). **Christine F. Baes:** supervision (supporting), writing – review and editing (equal). **Bayode O. Makanjuola:** methodology (lead), supervision (lead), writing – review and editing (equal).

## Funding

Data collection was supported by a PhD studentship to E.A.A. by the Universities Federation for Animal Welfare. F.P. appreciates funding from FAPESP project #2018/13600‐0 and from the Svenska Forskningsrådet FORMAS grant #2021‐00532.

## Conflicts of Interest

The authors declare no conflicts of interest.

## Supporting information


**Figure S1:** (a–d) Manhattan plot for the genome‐wide association study of caudal hippocampal bipolar neuron density in H&N and Hy‐Line Brown laying hens. The *y*‐axis represents the −log_10_ (*p* value) for the association of each single nucleotide polymorphism (SNP) and the *x*‐axis represents chromosomal position. No SNPs reached the Bonferroni‐corrected significance threshold (horizontal dashed line). (a) CB: caudal bipolar; (b) CM: caudal multipolar; (c) RB: rostral bipolar; (d) RM: rostral multipolar.

## Data Availability

The datasets analyzed during this study are available from a previous study (Armstrong et al. 2022) in which their microbiome sequencing data can be accessed on the NCBI Sequencing Repository Archive regarding the BioProject Number PRJNA762251. A publicly accessible repository contains the other datasets that were generated and analyzed during the previous study (Armstrong et al. 2022) and can be found at: https://doi.org/10.25405/data.ncl.14135153.
