# Supplementary material for: Genome‐Wide Association Analysis of Hippocampal Neuroplasticity as an Indicator of Stress Responsiveness in Laying Hens (Gallus gallus domesticus)
Source: Anim Genet. 2026 Sep 10;57(5):e70202. doi: 10.1002/age.70202 (PMC13560938; doi:10.1002/age.70202)
Supplement: Supplementary file 1 [file AGE-57-0-s001.docx]

**SUPPLEMENTARY DATA**

Supplementary Figure 1a:

Supplementary Figure 1b:

Supplementary Figure 1c:

Supplementary Figure 1d:

Supplementary Figure 1a-d. Manhattan plot for the genome-wide association study of caudal hippocampal bipolar neuron density in H&N and Hy-Line Brown laying hens. The y-axis represents the -log10(p-value) for the association of each single nucleotide polymorphism (SNP) and the x-axis represents chromosomal position. No SNPs reached the Bonferroni-corrected significance threshold (horizontal dashed line). (a) CB: Caudal Bipolar; (b) CM: Caudal Multipolar; (c) RB: Rostral Bipolar; (d) RM: Rostral Multipolar.
